# Small-Baseline Approach for Monitoring the Freezing and Thawing Deformation of Permafrost on the Beiluhe Basin, Tibetan Plateau Using TerraSAR-X and Sentinel-1 Data

**DOI:** 10.3390/s20164464

**Published:** 2020-08-10

**Authors:** Jing Wang, Chao Wang, Hong Zhang, Yixian Tang, Xuefei Zhang, Zhengjia Zhang

**Affiliations:** 1 Key Laboratory of Digital Earth Science, Aerospace information Research Institute, Chinese Academy of Sciences, Beijing 100094, China; wangjing2018@radi.ac.cn (J.W.); zhanghong@radi.ac.cn (H.Z.); tangyx@radi.ac.cn (Y.T.); zhangxf@radi.ac.cn (X.Z.); 2 University of Chinese Academy of Sciences, Beijing 100049, China; 3 Faculty of Information Engineering, China University of Geosciences, 388 Lumo Road, Wuhan 430074, China; zhangzj@cug.edu.cn

**Keywords:** Qinghai–Tibet, the active layer, NSBAS, freeze–thaw cycles, seasonal deformation

## Abstract

The dynamic changes of the thawing and freezing processes of the active layer cause seasonal subsidence and uplift over a large area on the Qinghai–Tibet Plateau due to ongoing climate warming. To analyze and investigate the seasonal freeze–thaw process of the active layer, we employ the new small baseline subset (NSBAS) technique based on a piecewise displacement model, including seasonal deformation, as well as linear and residual deformation trends, to retrieve the surface deformation of the Beiluhe basin. We collect 35 Sentinel-1 images with a 12 days revisit time and 9 TerraSAR-X images with less-than two month revisit time from 2018 to 2019 to analyze the type of the amplitude of seasonal oscillation of different ground targets on the Beiluhe basin in detail. The Sentinel-1 results show that the amplitude of seasonal deformation is between −62.50 mm and 11.50 mm, and the linear deformation rate ranges from −24.50 mm/yr to 5.00 mm/yr (2018–2019) in the study area. The deformation trends in the Qinghai–Tibet Railway (QTR) and Qinghai–Tibet Highway (QTH) regions are stable, ranging from −18.00 mm to 6 mm. The InSAR results of Sentinel-1 and TerraSAR-X data show that seasonal deformation trends are consistent, exhibiting good correlations 0.78 and 0.84, and the seasonal and linear deformation rates of different ground targets are clearly different on the Beiluhe basin. Additionally, there are different time lags between the maximum freezing uplift or thawing subsidence and the maximum or minimum temperature for the different ground target areas. The deformation values of the alpine meadow and floodplain areas are higher compared with the alpine desert and barren areas, and the time lags of the freezing and thawing periods based on the Sentinel-1 results are longest in the alpine desert area, that is, 86 days and 65 days, respectively. Our research has important reference significance for the seasonal dynamic monitoring of different types of seasonal deformation and the extensive investigations of permafrost in Qinghai Tibet Plateau.

## 1. Introduction

The total area of permafrost on the Qinghai–Tibet plateau (QTP) is approximately 1.06 ± 0.09 km^2^ [1], which is the largest area of high-altitude discontinuous permafrost in the middle and low latitudes of the world. Permafrost is an important component of the cryosphere and is sensitive to climate changes [2]. In the last 50 years, with global warming, permafrost on the QTP has been characterized by increasing temperatures, thickening of the active layer, shrinking, and thinning in permafrost areas [3]. The uppermost layer of the permafrost is the active layer, which thaws in summer and freezes in winter [4]. The active layer can cause seasonal uplift and subsidence of the surface during freeze–thaw cycles. These phenomena will have important impacts on the formation of underground ice and organic carbon, water resources, ecology and even the global climate system in permafrost areas [5]. Additionally, the freeze–thaw cycles of the frozen soil in permafrost areas can lead to extensive surface deformation and also seriously affects human engineering activities and infrastructure, such as the Qinghai–Tibet Railway (QTR) and Qinghai–Tibet Highway (QTH) [6,7,8]. Thus, it is highly important to monitor the surface deformation of permafrost on the QTP. Traditional methods of deformation monitoring in frozen areas include drilling [9], Global Positioning System (GPS) [10], leveling, and ground penetrating radar (GPR) [11]. However, these measurements are usually sparse and unevenly distributed.

In recent years, Synthetic Aperture Radar interferometry (InSAR) has begun to provide an opportunity to retrieve the surface deformation of the frozen soil on the QTP with the development of multiple, high resolution sensors with wide coverage and frequent acquisitions [12,13,14]. In the past two decades, by overcoming those limitations—such as temporal and geometrical decorrelations [15] and atmospheric delay anomalies [16]—time-series InSAR technology (TSInSAR) has been widely applied to monitor the freezing and thawing processes of the active layer in permafrost areas [14,17,18,19,20,21,22,23,24]. 

TSInSAR algorithms include Permanent Scatterer (PSInSAR^TM^) [25], Interferometric Point Target Analysis (IPTA) [26], Stanford Method for Persistent Scatterers (StaMPS) [27], Quasi-Persistent Scatterers (QPS) [28], Persistent Scatterer Pairs (PSP) [29], and Spatio-Temporal Unwrapping Network (STUN) [30]. To increase the spatial density of measurement points in rural areas, a new generation time series InSAR technique has been developed based on the combined analysis of permanent scatterers and distributed scatterers [31,32,33,34]. Initially, such scholars as Chen et al. and Xie et al. [6,17] employ the linear deformation model of PSInSAR technology to retrieve the deformation law of the frozen soil. However, this deformation model has some limitations; for example, the freezing and thawing processes of the frozen soil are affected by many factors (e.g., temperature, precipitation, water content, heat flow, and soil lithology), and the surface deformation trend is not linear [19]. Then Chen et al. propose the use of a cubic function to describe the seasonal deformation law of the frozen soil [35]. Based on the Stefan model, Liu et al. set up a seasonal deformation model that is related to the square root of the accumulated degree days of thaw (ADDT; °C days) to calculate the seasonal subsidence while hypothesizing that the seasonal subsidence is caused by thaw settlement of the active layer and that the secular subsidence is probably due to thawing of an ice-rich layer near the permafrost table [18,36]. Yuan [37] also uses a similar deformation model to retrieve the deformation of permafrost in the northeast of Greenland, and some researchers suggest that sinusoidal models are capable of approximating and effectively modeling the ground surface deformation of the frozen soil [20,21,38,39]. However, the seasonal deformation of the freeze–thaw cycles of permafrost in reality is susceptible to the influence of precipitation, temperature and other factors, which may not vary in accordance with the sinusoidal model. Zhao et al. propose a new deformation model considering both the external (mainly climatic) and internal factors (such as tectonic activities and the thermal character of frozen soil) [19]. Hu and Chen [10,23] incorporate a piecewise elevation change model that includes periodic subsidence/uplift as its seasonally varying components, as well as linear subsidence trends. In contrast, high resolution X-band TerraSAR-X images can explain the deformation features of different ground objects in more detail [22,40,41], and L-band ALOS-2 PALSAR images with longer wavelengths and better coherence avoid geometric decorrelation, significantly improving measurement accuracy for frozen soil areas [19,20,21]. ESA Sentinel-1A and B satellites provide an opportunity to monitor the surface deformation of permafrost for a large area [14,39,42,43]. To make the InSAR deformation model more suitable for the deformation law of frozen soil and to capture the dynamic process of different ground types on the QTP, we propose a seasonal deformation model based on this model by Hu et al. and Chen et al. [10,23].

In this paper, this seasonal deformation model is introduced to implement the NSBAS process and to retrieve the surface deformation related to the freeze–thaw cycles of permafrost using Sentinel-1 and TerraSAR-X data at different scales over the QTP. This model includes the seasonally varying components (subsidence/uplift), as well as linear and residual deformation trends, based on the accumulated degree-days of freeze and thaw. Comparing the results of two sensors, the difference in the deformation results and the deformation law of different typical ground targets on the Beiluhe basin are explained in more detail. Spatiotemporal analysis is implemented to analyze the correlation between seasonal deformation, linear deformation rates, slope, and elevation in the area of interest. In addition, the daily air temperature dataset combined with the deformation results are used to analyze the whole freeze–thaw cycles, and the relationship between the seasonal deformation and the active layer thickness are also analyzed based on GPR data.

## 2. Study Area and Datasets

### 2.1. Study Area

**Figure 1:**
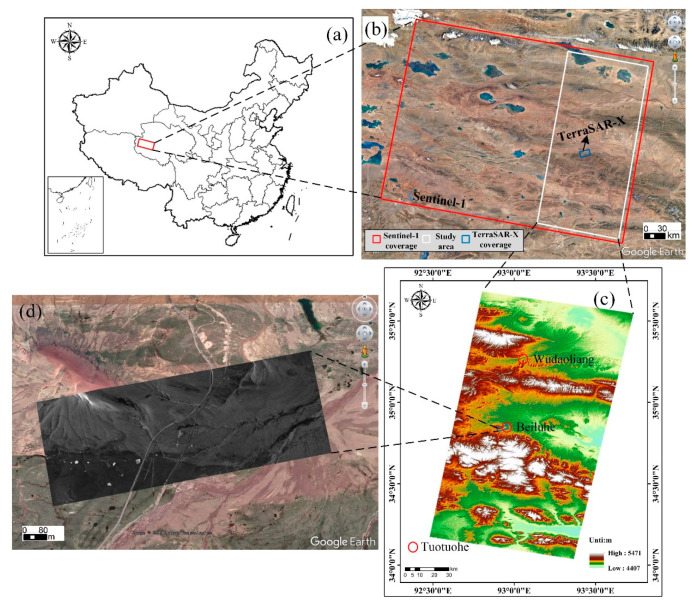
(**a**) Geographic location of the study area. (**b**) Coverage of SAR images and the study area. The red box indicates the coverage of Sentinel-1 data. The blue box indicates the coverage of TerraSAR-X data, and the white box represents the overlay area of the study area. (**c**) Topographic map, which is extracted from a Shuttle Radar Topography Mission Digital Elevation Model (SRTM DEM). (**d**) TerraSAR-X amplitude image in the blue box (Figure 1c).

The study area covering the Wudaoliang to Tuotuohe region is located in the northeast of the continuous permafrost zone on the QTP, belonging to Qinghai Province, China (Figure 1). Figure 1b shows the coverage of Sentinel-1 and TerraSAR-X images, and the white box represents the overlay area of the study area. Figure 1c shows that the study area is characteristic of a rolling topography with an elevation range from 4407 m to 5471 m. Figure 1d shows the TerraSAR-X amplitude image. According to Figure 1b,d and the field photos of the study area [39,42], the region is classified into six typical ground types: QTH Region, QTR Region, alpine desert, alpine meadow, barren, and floodplain, which is characterized by a sub frigid semiarid climate with an average temperature of approximately −3.8 °C [39,44]. The maximum and minimum temperatures in the study area are 23 °C and −38 °C, respectively. The frozen soil of the Beiluhe basin is characterized by ice-rich (volumetric ice content >25%), warm (mean annual ground temperature > −1.0 °C) permafrost. More than 90% of precipitation falls between May and September [45]. The active-layer thickness (ALT) ranges from 1.6 to 3.4 m [46]. The mean annual precipitation ranges from 300 to 400 mm, 80% of which is concentrated in the thawing period [47]. 

### 2.2. Datasets

In this paper, 35 scenes of C-band Sentinel-1 images with a 12 days revisit time from 7 August 2018 to 25 October 2019 and 9 X-band TerraSAR-X images with less than two month of revisit time from 15 December 2018 to 8 October 2019 were collected to implement the NSBAS process. Sentinel-1 is a two-satellite constellation, i.e., Sentinel-1A and Sentinel-1B, which were launched by European Space Agency (ESA) [48]. Sentinel-1 provides C-band SAR data with further enhancements in terms of, for instance, repeat cycle and coverage. Sentinel-1 images were captured along the N. 150 descending track with the Interferometric Wide swath (IW) TOPS mode and a 250 km swath. The coverage of TerraSAR-X images was approximately 2.8 × 7.5 km^2^, and TerraSAR-X data were acquired by the staring spotlight (ST) mode along the N. 385 ascending track. Detailed information is shown in Table 1.

In addition, the one arc-second SRTM DEM (pixel size of 30 m) was adopted to remove the topographic phase. Additionally, we obtained the 2−m air temperature in our study area by downscaling the ERA5 monthly averaged reanalysis product. These measurements can be downloaded from ERA-5 repository of European Centre for Medium-Range Weather Forecasts [49]. In this study, a field measurement was conducted in August 28, 2018 on the Beiluhe basin to detect shallow subsurface conditions in permafrost [50,51] and retrieve the ALT [52,53] by using LTD-2100 GPR( produced by China Research Institute of Radio Wave Propagation (CRIRP) in Qingdao, China ) [54]. 

## 3. Methodology

**Figure 2:**
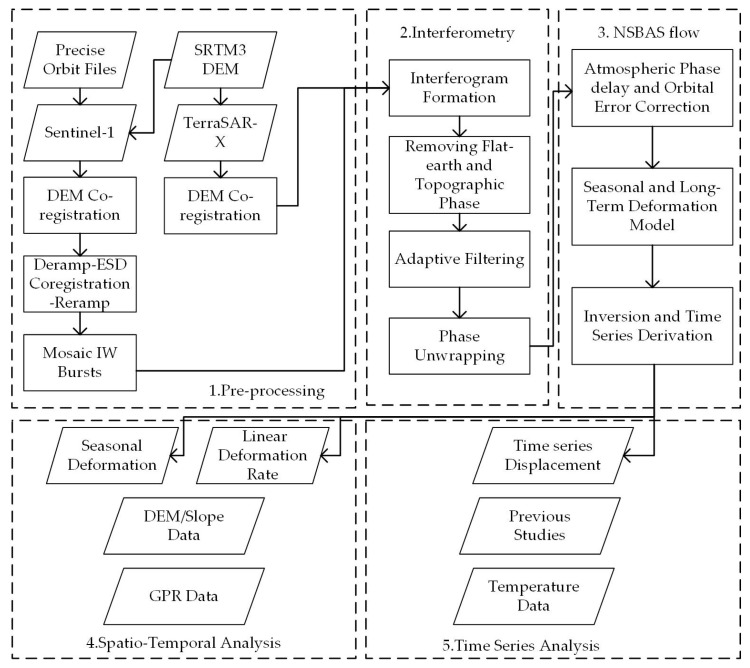
Flowchart of the NSBAS method of deformation analysis with the main processing steps.

In this study, we employed the NSBAS approach based on the Stefan model [10,23] to investigate the surface deformation of the study area in the freezing and thawing periods. The methodology includes the following four steps (Figure 2): (1) Sentinel-1 and TerraSAR-X preprocessing, including sub steps (Digital Elevation Model (DEM) and Enhanced Spectral Diversity (ESD) co-registration and mosaic bursts); (2) interferometry, including sub steps (interferogram formation, removing flat-earth and topographic phase, adaptive filtering, and phase unwrapping); preprocessing and interferometry process of TerraSAR-X data were implemented using SNAP software ( produced by European Space Agency (ESA) in Paris, France), while GMTSAR software (produced by Scripps Institution of Oceanography in California, USA) was used in Sentinel-1 data preprocessing and interferometry processes [55]. (3) NSBAS flow, in which we modified the linear deformation model to the seasonal and long-term deformation model; time series deformation inversion and the NSBAS flow were completed using the GInAT software ( supported by NASA solid earth and natural hazards program (NNX09AD25G), the Keck Institute for Space Studies (KISS) and the Caltech Tectonics Observatory (CTO) in USA ) [56,57]; (4) Spatiotemporal analysis, in which the relation between the seasonal deformation and the DEM\slope and GPR data were analyzed; and (5) time series analysis. 

### 3.1. Sentinel-1 and TerraSAR-X InSAR Processing

**Figure 3:**
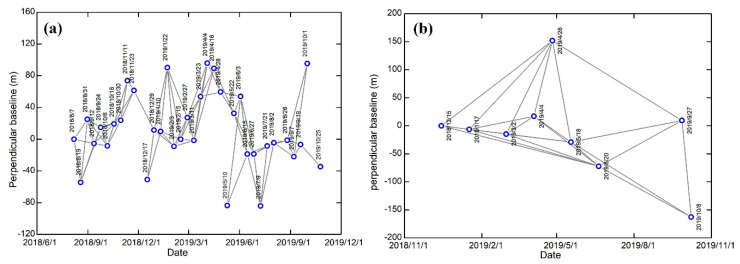
Spatial and temporal baselines of the interferograms. (**a**) Sentinel-1 data. (**b**) TerraSAR-X data.

The DEM co-registration method was adopted for TerraSAR-X and Sentinel-1 data. Furthermore, the ESD method was used to refine the azimuth offset of the Sentinel-1 images [58]. Additionally, deramping, interpolation, and reramping were performed on each burst [59], and then all the bursts were mosaicked. The blue circles in Figure 3a,b represent the acquisition dates of the SAR images, and the lines represent a set of interferograms. The perpendicular and temporal baselines of Sentinel images are less than 100 m and 50 days, and TerraSAR-X images set a suitable threshold for the 180 m spatial baselines and 187 day temporal baselines. Additionally, some interferograms were removed due to serious decorrelation and atmospheric phase delay. Finally, 80 Sentinel-1 interferograms and 23 TerraSAR-X interferograms were selected, as shown in Figure 3. Flat-earth and topographic phase were removed using the 3 arc-second SRTM DEM. To increase the signal-to-noise ratio of each interferogram, all the Sentinel-1 interferograms were multilooked by factors of 8 and 2 along the range and azimuth directions, respectively, TerraSAR-X interferograms were multilooked by a factor of 2 in range direction and of 12 in the azimuth direction. Then, a nonlinear adaptive spatial filtering was applied to each interferogram [60]. The minimum cost flow (MCF) algorithm was used to perform phase unwrapping [61]. Then, the fifth generation European Centre for Medium-Range Weather Forecasts (ECMWF) reanalysis for the global climate ERA5 [49] was used to estimate atmospheric phase using a stand-alone Python module, PyAPS (Python-based Atmospheric Phase Screen), in the Generic InSAR Analysis Toolbox (GIAnT) toolbox, and atmospheric corrections were implemented for all the unwrapped interferogram, which can also correct residual orbit errors from each interferogram by network deramping methods. Finally, we obtained the seasonal deformation changes and time series displacements using the NSBAS inversion. 

### 3.2. Seasonal and Long-Term Deformation Model

**Figure 4:**
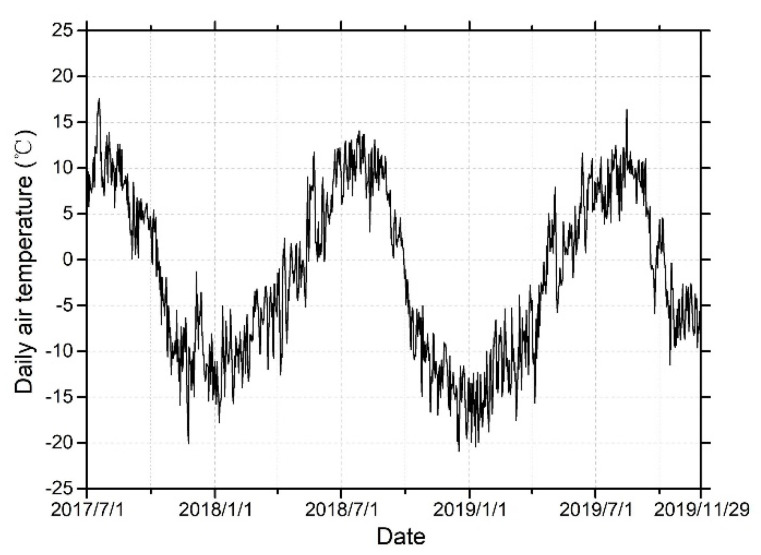
2-m air temperature data in this study area from 7/1/2017 to 11/29/2019.

Permafrost areas generally experience seasonal uplift and subsidence. The linear deformation model is not suitable to retrieve the freeze–thaw cycle deformation of the frozen soil [20]. In this study, we used a piecewise deformation change model, including seasonal and long-term deformation, by integrating the complete freeze–thaw cycles proposed by Hu [10], and we modify this model and add residual deformation. This observed deformation during the SAR images acquisition time can be summed across the seasonal and the linear deformation changes [23] in Equation (1).

(1) $$D(t)=\{\begin{array}{cc} V\cdot (t-t_{T})+S\cdot I(t)+c & \;\;if\;\;t_{T}\leq t\leq t_{F}\\ V\cdot (t-t_{F})+S\cdot I(t)+c & \;\;if\;\;t_{F}\leq t\leq t_{T} \end{array}$$

where D(t) is the total surface deformation on any day t since the onset of thawing or freezing; V is the linear deformation rate (mm/yr); $t_{T}$ and $t_{F}$ are the onsets of thawing and freezing, which can be obtained based on daily air temperature from Figure 4; S is the seasonal deformation rate (mm); c is the residual deformation between the observations and the fitted model; and I(t) is the composite index to combine the thaw and freeze indices in Equation (2).

(2) $$I(t)=\sqrt{ADDT(t)}-\sqrt{\frac{k_{F}n_{F}}{k_{T}n_{T}}}\sqrt{ADDF(t)}$$

where ADDT(t) and ADDF(t) are the square root of the accumulated degree days of thaw (*ADDT*) and freeze (*ADDF*) to account for the seasonal thaw subsidence and freeze uplift, respectively, and they are calculated based on the 2-m air temperature by Liu et al [18]. $k_{F}=1.4\;W\cdot M^{-1}\cdot K^{-1}$ and $k_{T}=0.6\;W\cdot M^{-1}\cdot K^{-1}$ are the soil thermal conductivity that can be obtained from the local soil type and volumetric water content [62]. $n_{F}=0.61$ and $n_{T}=0.62$ were calculated by Cao et al. [63].

### 3.3. NSBAS Method Based on the Seasonal and Long-Term Deformation Model

The gradual deformation change (subsidence/uplift) is primarily associated with the freezing and thawing of the active layer and the uppermost permafrost on the Beiluhe basin. Therefore, we adopted the NSBAS method based on the deformation model, including seasonal and long-term deformation changes, to retrieve the surface deformation of freeze–thaw cycles. The seasonal and long-term deformation model is shown in Equation (3)

(3) $$\Delta \varphi_{m}^{}=\Delta \varphi_{i}^{s+l}+\Delta \varphi_{i}^{topo\_res}+\Delta \varphi_{i}^{res}=\frac{4\pi }{\lambda }(D(t)+\frac{B_{⊥}\Delta \varepsilon }{R\sin\theta })+\Delta \varphi_{i}^{res}$$

where λ is the radar wavelength; i is the number of unwrapped interferograms; $\Delta \varphi_{m}^{}$ denotes the model phase; $\Delta \varphi_{i}^{s+l}$ is the seasonal and long-term deformation phase; $\Delta \varphi_{i}^{topo\_res}$ represents the phase contribution related to DEM error, which be related to the perpendicular baseline $B_{⊥}$, slant range distance R and incidence angle θ; D(t) is the observed deformation in Equation (3); and $\Delta \varphi_{i}^{res}$ is the residual phase, including phase noise, atmospheric delay, and orbit error phase.

In the traditional SBAS approach, the set of interferometric phase observations is written as a linear combination of individual SAR scene phase values for each pixel independently [33] in Equation (4).

(4) $$d_{k}^{l}=G_{k}^{l}\cdot m_{k}^{l}\Leftrightarrow \Phi_{i,j}=\sum_{n=i}^{j-1}\delta \varphi_{n}$$

where l is a specific pixel; k∈(1,M); M is the number of interferograms; $d_{k}^{l}$ represents a data vector composed of the interferometric phase; $G_{k}^{l}$ is a matrix of zeros and ones directly related to the set of interferograms generated from the available data; $m_{k}^{l}$ is the phase delay increment; $\Phi_{i,j}$ is the pixel phase value for the interferogram combining acquisition of i and j; and $\delta \varphi_{n}$ is the phase increment between acquisition *n* and *n* + 1. Singular value decomposition (SVD) is used instead of least squares to retrieve the deformation time series in Equation (4). 

However, $G_{k}^{l}{}^{T}G_{k}^{l}$ has a rank deficiency, and the incremental phase delay between successive images groups is set to zero by SVD [33]. To overcome this SVD problem, the NSBAS method adds constraints to the inversion, which was proposed by Doin et al. [64] as

(5) $$\forall d\in [2,N]\begin{array}{cc} & \sum_{n=1}^{d-1}\delta \varphi_{n}-D(\Delta t)+eB_{⊥}^{d}=0 \end{array}$$

where N denotes acquisitions dates; $B_{⊥}^{d}$ s the perpendicular baseline between satellite paths at acquisition 1 and d; and D(Δt) is used as a regularization function as discussed in Equation (1) and is a parametric representation of the temporal form of the deformation. Then, V and S are estimated for each pixel from the set of interferograms by the least squares method in the constrained linear system. Additionally, the NSBAS method takes advantage of a user-defined functional form of the phase evolution to overcome the issue of missing links in the interferometric network due to temporal and spatial decorrelation.

In this study, GIAnT software was used to implement the NSBAS process [56]. Finally, the seasonal deformation, the linear deformation changes and the residual deformation were estimated, while the time-series deformations were also obtained.

## 4. Results and Analysis

### 4.1. InSAR Results 

The proposed deformation model of Section 3.2 based on the Sentinel-1 data was used to estimate the amplitude of seasonal deformation, linear deformation rate and residual deformation from Wudaoliang to Tuotuohe. Meanwhile, TerraSAR-X and Sentinel-1 InSAR results were also used to further analyze the deformation trends of different ground targets on the Beiluhe basin.

#### 4.1.1. Sentinel-1 Results From Wudaoliang to Tuotuohe

**Figure 5:**
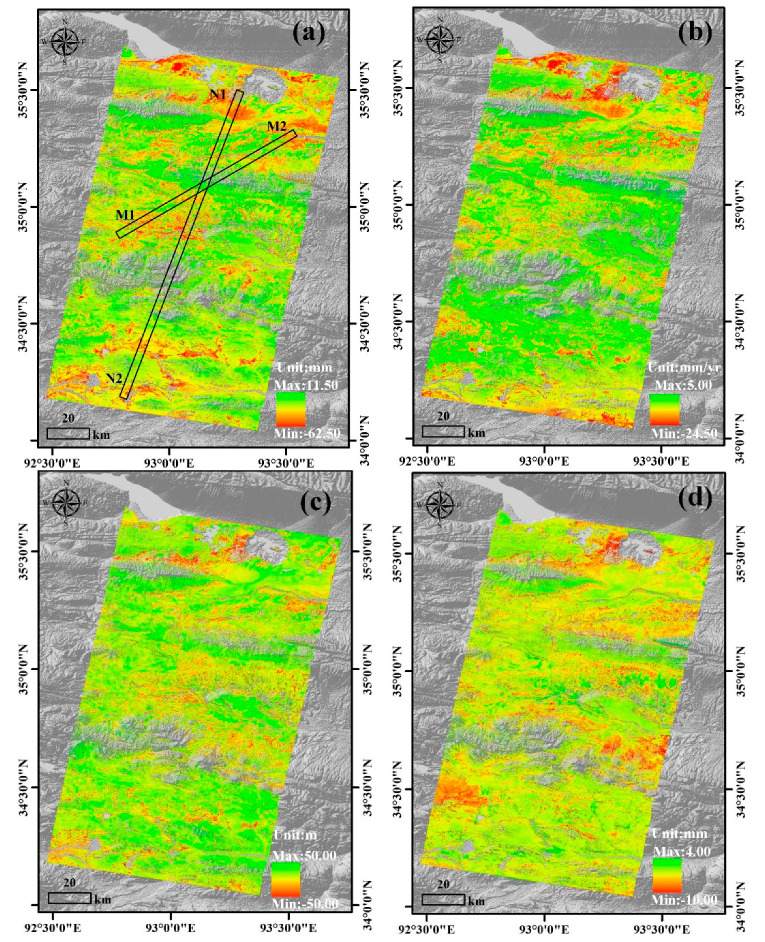
Sentinel-1 InSAR results based on the seasonal and long-term deformation models over 08/07/2018–10/25/2019. (**a**) Amplitude of seasonal deformation. (**b**) Linear deformation rate. (**c**) DEM error. (**d**) Residual deformation.

The Sentinel-1 InSAR results from Wudaoliang to Tuotuohe are shown in Figure 5. Figure 5a depicts the amplitude of the seasonal deformation map. The overall seasonal deformation ranges from −62.50 mm to 11.50 mm in the study area and is generally smaller than 50 mm in most areas. The seasonal deformation was mainly caused by the freezing uplift and thawing subsidence of the active layer, and the variation is caused by the different thickness of the active layer [39]. The higher values of seasonal deformation are mainly distributed on the Beiluhe basin because the active layer is thicker and is dominated by subsandy soil and silty clay with rich water content. Figure 5b shows that the linear deformation trends are up to −24.50 mm/y, and the linear subsidence values in most areas are approximately 10 mm/yr. The DEM error inferred from Figure 5c ranged from −50 m to 50 m, which is similar to the seasonal deformation trend. Figure 5d shows that the range of residual deformation is between −10.00 mm and 4.00 mm.

#### 4.1.2. Sentinel-1 and TerraSAR-X InSAR Results on the Beiluhe Basin

**Figure 6:**
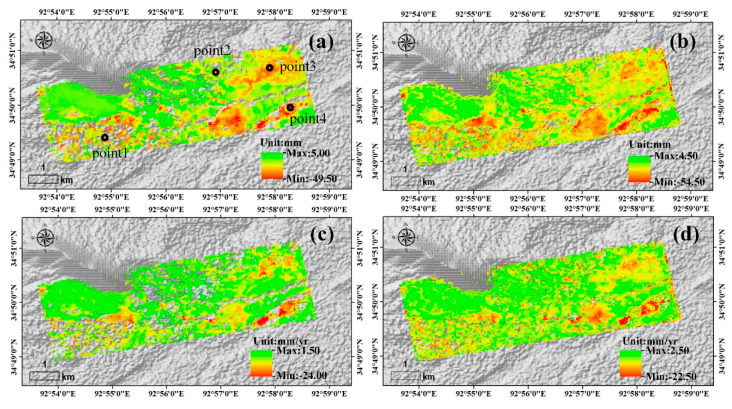
Sentinel-1 and TerraSAR-X InSAR results in the Beiluhe basin. (**a**) Amplitude of seasonal deformation of Sentinel-1 during the period 08/07/2018–10/25/2019. (**b**) Amplitude of seasonal deformation of TerraSAR-X during the period 12/15/2018–10/08/2019. (**c**) Linear deformation rate of Sentinel-1 during the period 08/07/2018–10/25/2019. (**d**) Linear deformation rate of TerraSAR-X during the period 12/15/2018–10/08/2019.

The Beiluhe basin is located in the middle of our study area. Figure 6 shows the amplitudes of seasonal deformation and the linear deformation rates results of Sentinel-1 and TerraSAR-X data on the Beiluhe basin. Compared to the longer revisit time and X-band of TerraSAR-X [65], the C-band and a 12-day revisit time of Sentinel-1 data provides less decorrelation and better coherence on wet and fast-moving permafrost surfaces. The seasonal deformation and linear deformation rates on the Beiluhe basin from TerraSAR-X data range from −54.5 mm to 4.5 mm and −22.5 mm/yr to 2.5 mm/yr, respectively, and those from Sentinel-1 data range from −49.5 mm to 5.0 mm and −24.0 mm/yr to 1.5 mm/yr, respectively. The maximum amplitude of seasonal deformation of TerraSAR-X images is greater than that of the Sentinel-1 images, and there is a subtle difference between the linear deformation rate and the seasonal deformation of the different datasets in some regions due to the different observation periods of the two sensors. However, the overall deformation trends of the seasonal deformation and linear deformation rate of the two datasets are consistent. 

### 4.2. Spatiotemporal Analysis of Deformation Results 

**Figure 7:**
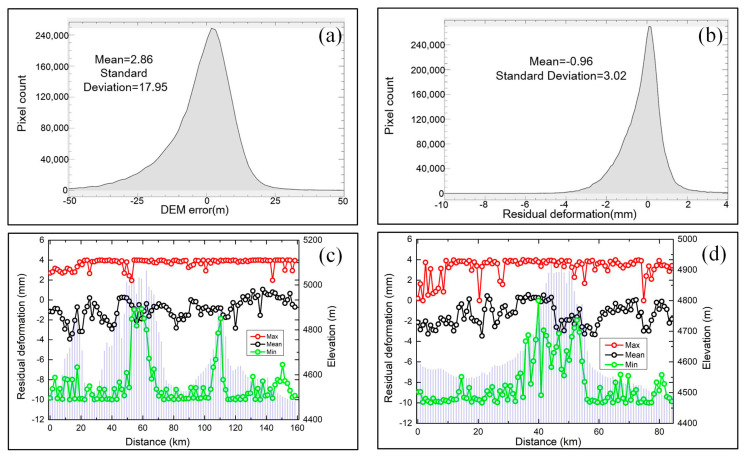
Relation between the maximum (red), minimum (green), and mean (black) value and the elevation (blue) of the residual deformation. (**a**) Histogram of the DEM error. (**b**) Histogram of the residual deformation. (**c**) N1-N2 profile. (**d**) M1-M2 profile.

To investigate the spatial distribution characteristics and deformation trends of the InSAR results, we extracted the N1-N2 and M1-M2 profiles from the amplitude of seasonal deformation, linear deformation rate, and residual deformation maps (Figure 5). We divided the N1-N2 and M1-M2 profiles into 100 subunits and extracted the minimum, maximum and mean values of each subunit for detailed analysis. Figure 7a shows that the standard deviation and mean value of the DEM error are 17.95 mm and 2.86 mm, respectively. Figure 7b shows the residual deformation map with a mean of −0.96 mm and standard deviation of 3.02 mm. Figure 7c,d show that the mean residual deformation of the N1-N2 and M1-M2 profiles are between −4 mm and 1 mm, and the minimum value ranges from −10 mm to 0 mm. The residual deformation changes may be related to the orbit errors and residual DEM errors. Due to the residual deformation and DEM error with small dispersion, the deformation results are reliable based on the seasonal and long-term deformation model.

**Figure 8:**
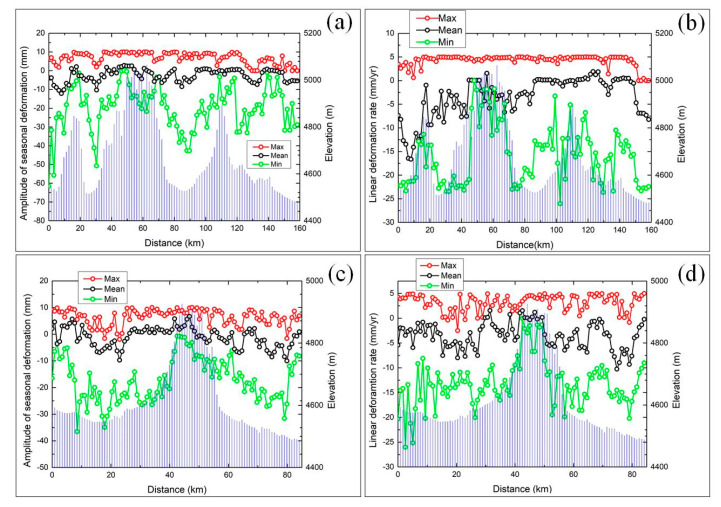
Relation between the maximum (red), the minimum (green), mean (black) value and elevation (blue) of the amplitude of the seasonal deformation and linear deformation rate. (**a**) Amplitude of the seasonal deformation profile along N1 to N2. (**b**) Linear deformation rate profile along N1 to N2. (**c**) Amplitude of the seasonal deformation profile along M1 to M2. (**d**) Linear deformation rate profile along M1 to M2.

**Figure 9:**
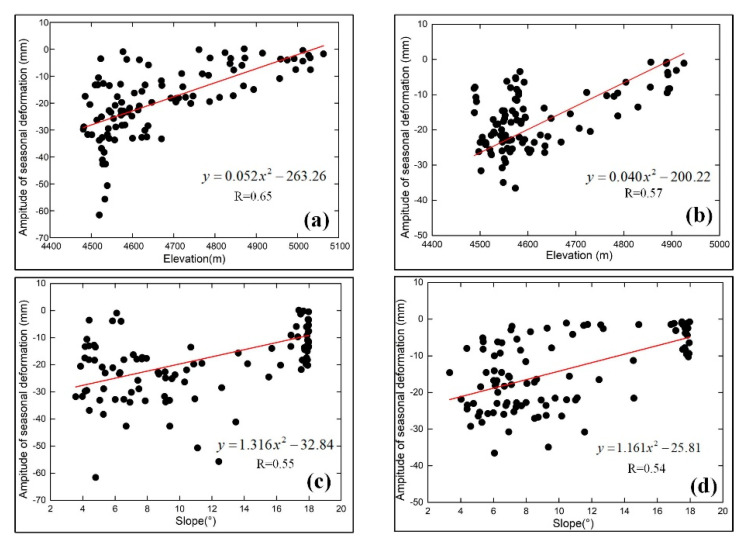
Correlations between the height, the slope and amplitude of the seasonal deformation. (**a**) Height and amplitude of the seasonal deformation profile along N1 to N2. (**b**) Height and amplitude of the seasonal deformation profile along M1 to M2. (**c**) Slope and amplitude of the seasonal deformation profile along N1 to N2. (**d**) Slope and amplitude of the seasonal deformation profile along M1 to M2.

Figure 8 shows that the maximum, minimum and mean values of the amplitude of the seasonal and linear deformation rate are negatively correlated with elevation. As the elevation increases, the annual average temperature decreases, the ALT which is primarily affected by soil properties and soil moisture decreases, and the deformation trends decrease accordingly [43]. In contrast, solid precipitation (snow) in these high-altitude areas can only lead to small changes in the soil moisture content; thus, the hydrothermal properties remain stable, and the deformation values are relatively low. In addition, because an increase in slope leads to elevation increases. Figure 9 shows a subtle negative correlation between the height, the slope and amplitude of seasonal deformation. The correlation coefficients of the height and amplitude of seasonal deformation{n along the N1-N2 and M1-M2 profiles are, respectively, 0.65 and 0.57 (Figure 9a,c), and the weak correlation coefficients of the slope and amplitude of seasonal deformation rate along the N1-N2 and M1-M2 profiles are, respectively, 0.55 and 0.54 (Figure 9b,d).

**Figure 10:**
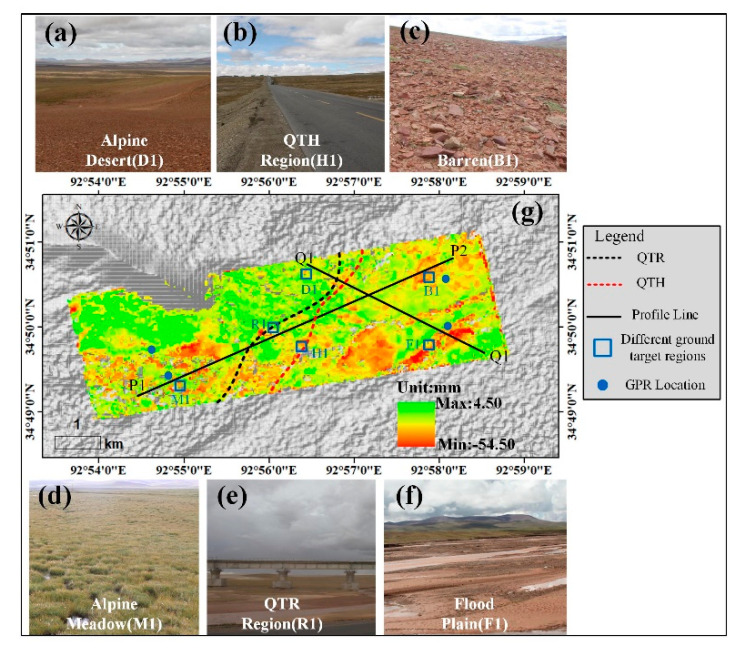
Field photos of typical ground targets and the TerraSAR-X seasonal deformation map (**a**) Alpine Desert (D1). (**b**) QTH Region (H1). (**c**) Barren (B1). (**d**) Alpine Desert (D1). (**e**) QTR region (R1) (**f**) Floodplain (F1). (**g**) TerraSAR-X amplitude map.

To analyze the deformation trends of the different ground targets in more detail, the landform of the Beiluhe basin was divided into six ground targets [41]: QTH Region, QTR Region, alpine desert, alpine meadow, barren, and floodplain based on the TerraSAR-X seasonal deformation map and the field investigation photos shown in Figure 10 (The investigation was conducted on 29 August 2018 on the Beiluhe basin). Figure 10g shows that these study areas with high seasonal deformation are mainly in the alpine meadow, floodplain, and alpine desert regions. The amplitude of the seasonal deformation ranges from -20 mm to 4 mm in the QTR and QTH regions. We extracted the amplitude of the seasonal deformation and linear deformation rates of profiles P1-P2 and Q1-Q2. 

**Figure 11:**
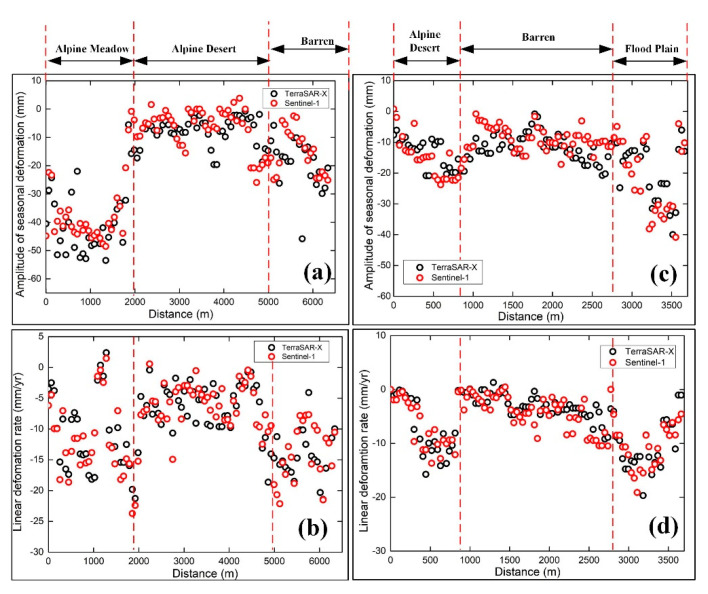
(**a**) Amplitude of the seasonal deformation along the P1-P2 profile. (**b**) Linear deformation rate along the P1-P2 profile. (**c**) Amplitude of the seasonal deformation along the Q1-Q2 profile. (**d**) Linear deformation rate along the Q1-Q2 profile.

**Figure 12:**
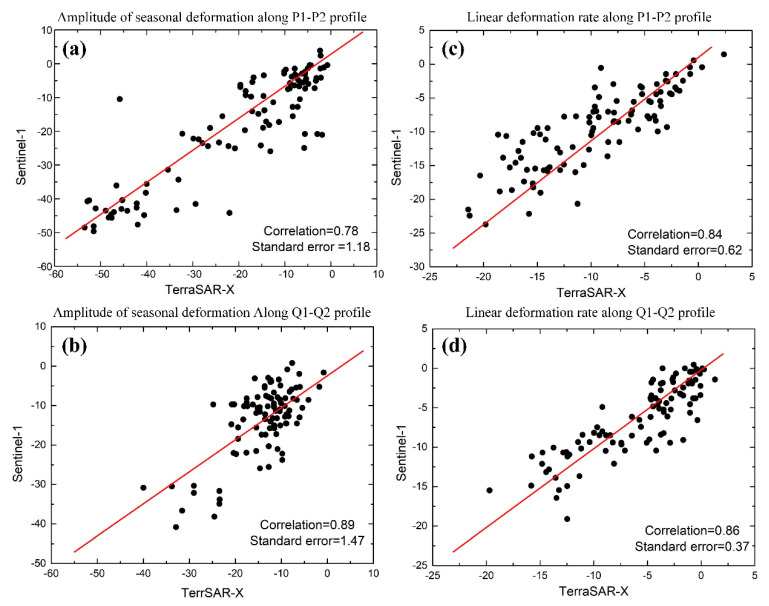
(**a**) Correlation of the amplitude of the seasonal deformation of TerraSAR-X and Sentinel-1 along the P1-P2 profile. (**b**) Correlation of the amplitude of the seasonal deformation of TerraSAR-X and Sentinel-1 along the Q1-Q2 profile. (**c**) Correlation of the linear deformation rate of TerraSAR-X and Sentinel-1 along the P1-P2 profile. (**d**) Correlation of the linear deformation rate of TerraSAR-X and Sentinel-1 along the Q1-Q2 profile.

Figure 11 shows the deformation profiles map of different ground targets for the P1-P2 and Q1-Q2 profiles from the two datasets in Figure 6, and Table 2 shows the ranges of amplitude of the seasonal deformation and linear deformation rates in different ground areas. Figure 11a,b show the significant differences in the amplitude of the seasonal deformation and linear deformation rates of the alpine meadow, alpine desert, and barren areas along the profile P1 to P2. The amplitude of the seasonal deformation of the Sentinel-1 data along the profile P1-P2 is up to −48.53 mm in the alpine meadow area, while the seasonal deformation of the TerraSAR-X data range from −53.49 mm to −5.54 mm. The amplitude of the seasonal deformation and linear deformation rate of the barren are relatively higher than that of the alpine desert because of the higher soil moisture content. Due to the higher soil temperature and moisture content, Figure 11c and Table 2 shows that the amplitude of the seasonal deformation along the profile Q1-Q2 in the floodplain area is high, with the TerraSAR-X data ranging from −39.98 mm to −6.05 mm and the Sentinel-1 data ranging from −38.17mm to −4.02 mm. Figure 11c,d show that there is no significant difference in the amplitude of the seasonal deformation and linear deformation rate between the alpine desert and barren area, but the linear deformation rate of TerraSAR-X in the floodplain area is up to −19.70 mm/y. To quantitatively evaluate the accuracy and reliability of the TerraSAR-X and Sentinel-1 results, we performed comparisons between deformations measured by the two sensors along the P1-P2 and Q1-Q2 profiles. Figure 12 shows the correlation and standard errors between the InSAR results for the TerraSAR-X data and Sentinel-1 data along the P1-P2 and Q1-Q2 profiles. The amplitude of the seasonal deformations and linear deformation rates of the two sensors along the P1-P2 profile showed good correlations of about 0.78 and 0.84, respectively (Figure 12a,b), while Figure 12c,d show the good correlations of the two InSAR results of 0.89 and 0.86, respectively. Due to the intrinsic properties of the two sensors and datasets (observation periods, temporal and spatial resolutions, sensor wavelengths, LOS, etc.) [14], there are some differences between the TerraSAR-X and Sentinel-1 results along the Q1-Q2 profiles, and these measurements along Q1-Q2 do not agree well with each other.

### 4.3. Time-Series Deformation Analysis 

**Figure 13:**
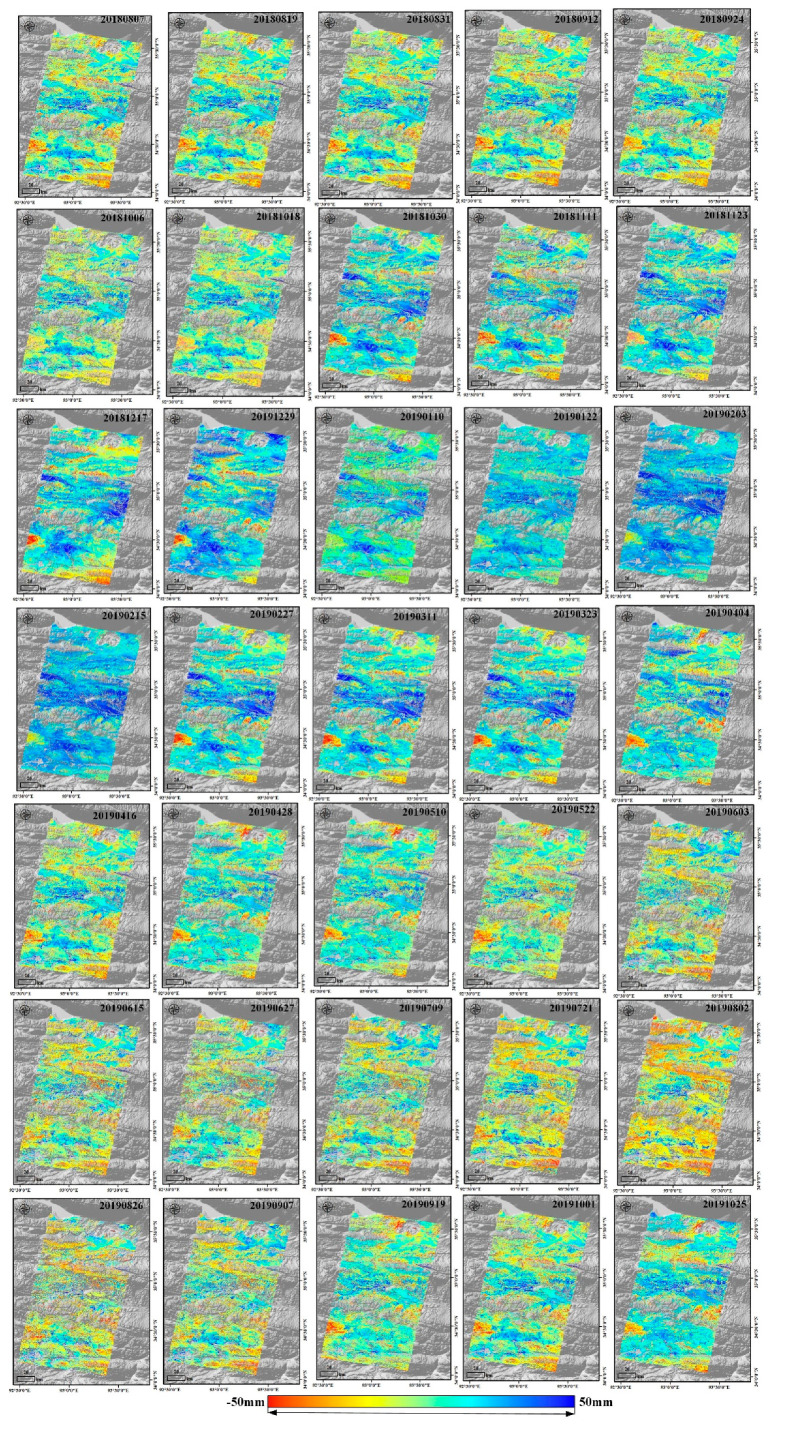
Time series deformation of the study area. The acquisition from 8 August 2019 is set as the reference image.

**Figure 14:**
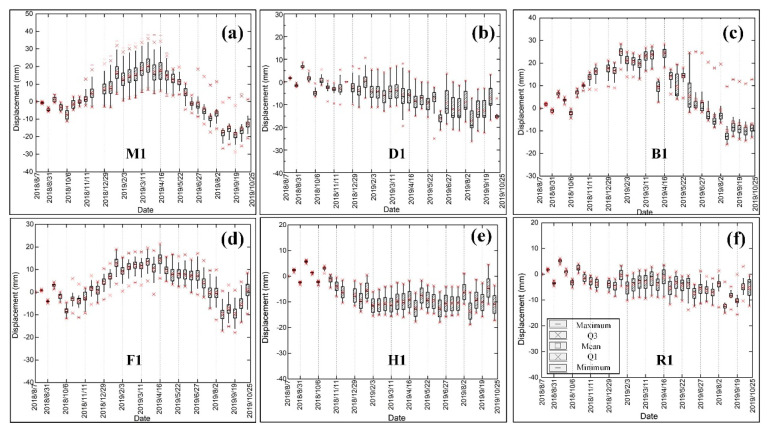
Sentinel-1 displacement time series at six locations with six typical ground target regions on the Beiluhe basin. (**a**) Alpine meadow. (**b**) Alpine desert. (**c**) Barren. (**d**) Floodplain. (**e**) QTH Region. (**f**) QTR Region.

Figure 13 shows the time series deformation map and the complete freeze–thaw cycles during 08/07/2018-10/07/2019 of Sentinel-1 data in the study area. The acquisition from the 7 August 2019 image is selected as the reference image. The frozen uplift reached the summit on 13 February 2018, while the thaw subsidence was up to 50 mm on 2 August 2019 in most areas. Permafrost was relatively stable from November to December each year in this region, and the freezing period lasted until February of the next year. From April to September 2019, with the temperature and the precipitation increasing, the heat of the active layer of the permafrost transferred from the top to the bottom, and the thawing process gradually began from the top of the active layer, causing a one-way thawing subsidence process of the active layer of the permafrost. Between the end of April and the beginning of May 2019, the temperature began to increase sharply. During the summer months of July and August, the temperature and precipitation reached extreme values for the region, and the subsidence also reached its maximum of 50 mm. Additionally, the time series deformation boxplots from six typical ground target regions (Figure 10) are plotted in Figure 14. There are obvious seasonal deformation trends in the alpine desert, alpine meadow, floodplain, and barren areas. Compared with the alpine meadow and floodplain, the displacement of the alpine desert and barren are lower, with mean displacement value ranging from −22.50 mm to 19.60 mm, which is consistent with the analysis in Section 4.2. The QTR and QTH regions have a small subsidence trend, with maximums of 15.57 mm and 18.23 mm, due to human activity, and the annual periodic displacement of these regions ranges from −18 mm to 6 mm.

### 4.4. Freeze–Thaw Cycles of Permafrost in the Beiluhe Basin 

**Figure 15:**
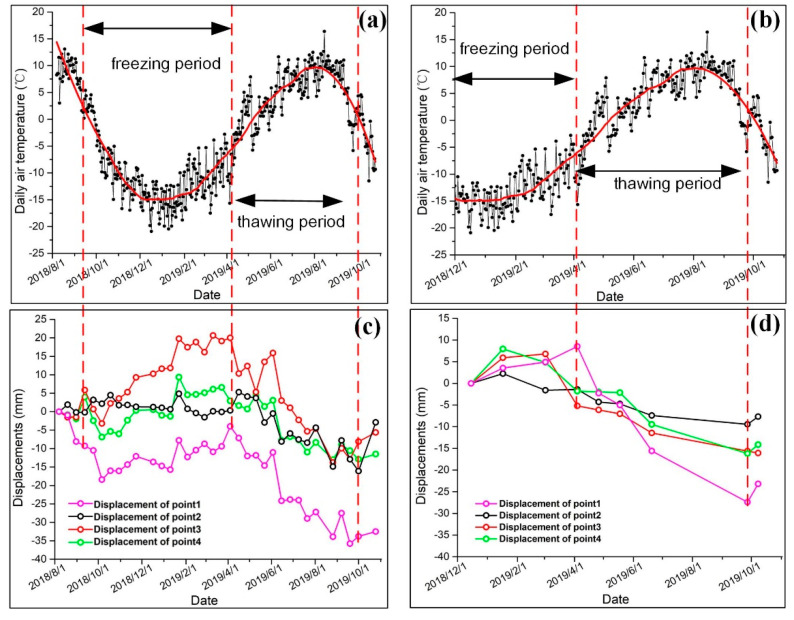
Daily air temperature (2 m surface) and NSBAS time series displacement of the alpine meadow (point 1) alpine desert (point 2), barren (point 3), and floodplain (point 4) during the freezing and thawing periods. (**a**) Daily air temperature from 8/1/2018 to 11/1/2019. (**b**) Daily air temperature from 12/1/2018 to 11/1/2019. (**c**) Sentinel-1 NSBAS time series displacement. (**d**) TerraSAR-X NSBAS time series displacement.

To study the freeze–thaw cycles of permafrost, we extracted the time series displacement maps of the four points marked in Figure 6a (point 1: alpine meadow, point 2: alpine desert, point 3: barren, point 4: floodplain) from Figure 13 for discussing the freeze–thaw process of the Beiluhe basin. Figure 15c,d shows the displacement changes of the Beiluhe basin during the freezing and thawing periods. Table 3 shows the time lags between the InSAR-observed displacement of the different ground points and the daily air temperature data. Due to the lack of TerraSAR-X images in August, all the time lags of different ground points in the thawing period based on TerraSAR-X data were 61 days. Figure 15a,b show that the time lags of the alpine desert based on TerraSAR-X and Sentinel-1 data are the longest, likely because the activity layer thickness of the alpine desert is thicker than that of other regions. The time lags from TerraSAR-X and Sentinel-1 data between the date of the maximum uplift and the date of the minimum temperature (01/20/2019) were 86 days and 96 days, respectively, and the time lags from between the date of the maximum subsidence and the date of the maximum temperature (07/28/2019) were 86 days and 96 days, respectively. The time lags between the temperature and the deformation of frozen soil in the present study are consistent with the conclusions of previous studies [13,20]. Although the surface temperature of permafrost may increase to its highest or fall to its lowest, the bottom of the active layer continues freezing or thawing until it reaches its maximum freezing or thawing depth after this time delay. 

The thawing process began from the surface of the frozen soil in mid-April in 2019, and it ended in mid-September in 2018 or 2019. The displacement maps of the Sentinel-1 and TerraSAR-X data in Figure 15c,d and Table 2 show that different ground points began to thaw from mid-March through April 2019. The thawing subsidence of the alpine desert is less than that of the alpine meadow and floodplain in summer, with maximum settlement of 14.86 mm and 16.20 mm for the Sentinel-1 and TerraSAR-X data, respectively. However, due to the rich soil water content, the deformation changes of the barren and floodplain regions are more drastic, and the subsidence is obvious. When the active layer reached its maximum thawing depth, this one-way freezing period began freezing from the bottom of the active layer and then froze gradually upwards. This period is the autumn freezing process in the Beiluhe area. Figure 15c,d show that different typical ground targets began to freeze from the end of September 2018 or the end of September 2019 followed by a two-way freezing process in the active layer from the surface of the frozen soil until the end of the freezing process was reached. After the freezing process of the active layer ended, the temperature began to drop rapidly, and the active layer was in a cooling process, which lasted until mid to late January of the next year (Figure 15a,b). From the end of March to the beginning of April 2019, the surface began to subside again. The above process is the complete freeze–thaw cycle for the Beiluhe area. 

## 5. Discussion

### 5.1. Comparison with Other Surface Subsidence Studies on the QTP

To verify the validity of our work, we compared our study with other studies on the QTP (Table 4). Li et al. [20] observed a seasonal amplitude of 0.5 mm–28 mm using a sinusoidal model. Wang et al. [8] reported amplitudes of seasonal deformation of 0–90 mm from 2014 to 2015 in the Beiluhe region. Daout et al. [12] reported seasonal oscillations ranging from 2.5 mm to 12 mm during 2003–2011 in the northwest of the QTP. Jia et al. [21] observed seasonal amplitudes of 0–20 mm using ALOS PALSAR data from 2007 to 2009. Chen et al. [23] incorporated a piecewise elevation change model that includes periodic subsidence/uplift and observed seasonal amplitudes of −60–60 mm during 2006–2011. Compared with our previous studies, Zhang et al. [39] employed a sinusoidal model and reported seasonal amplitudes of 0–30 mm from 2017 to 2018, but only Sentinel-1 data were adopted to investigate the surface deformation of the QTP, with wide coverage. This study based on Chen et al. [23] deformation model collected Sentinel-1 data with 12 days of revisit time and TerraSAR-X data with less than two months of revisit time and conducted more detailed deformation studies on different ground targets. Due to their different time periods and locations, there were some differences between these studies and our study. However, the trends of gradual subsidence were all on the order of centimeters per year. In addition, we used a similar model to Chen’s to retrieve seasonal and linear deformation, and thus the range of deformation that we observed was similar to Chen’s subsidence trends.

### 5.2. Analysis of InSAR Results and ALT Based on GPR Data 

**Figure 16:**
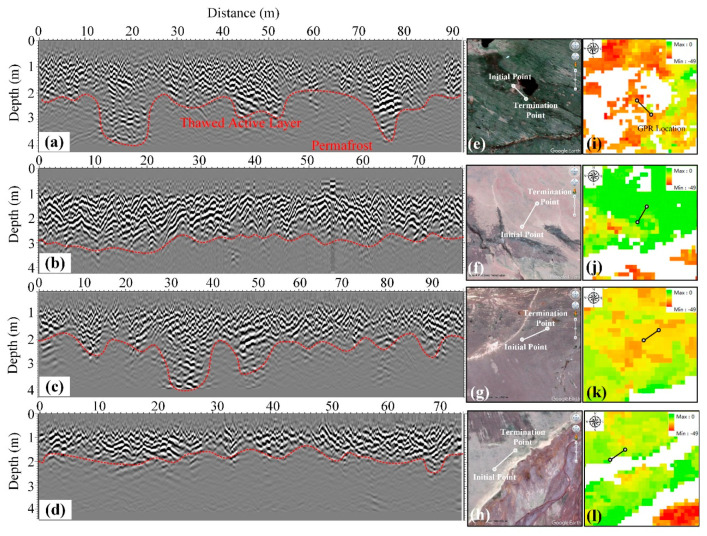
Interpreted GPR results of the ALT profile in Beiluhe basin. (**a**) Alpine meadow. (**b**) Alpine desert. (**c**) Barren. (**d**) Floodplain. (**e**–**h**) The site locations of GPR on Google earth images. (**i**–**l**) Amplitude of the seasonal deformation map of the Sentinel-1 data.

**Figure 17:**
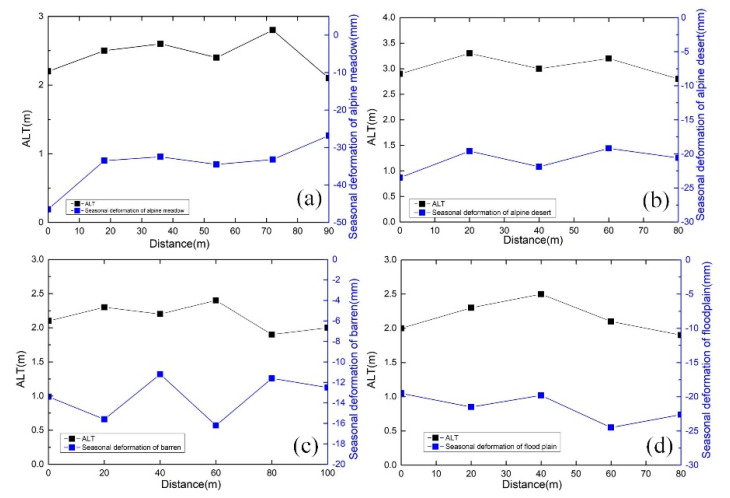
The relationship between amplitude of seasonal deformation and ALT. (**a**) Alpine meadow. (**b**) Alpine desert. (**c**) Barren. (**d**) Flood plain.

In Section 4.1, we demonstrated that there is a close correspondence between the amplitude of seasonal deformation and ALT and that ALT is also different at different altitudes. Our previous studies and other studies have established the relationship between amplitude of seasonal deformation and ALT [21,22,23,39,42]. We obtained the boundaries of the thawing layer of the alpine meadow, alpine desert, barren, and floodplain based on GPR data, and Figure 16 shows that the average thawing depths of the four typical ground targets are, respectively, 2.5, 3, 2.2, and 2 m. The ALT values of the alpine meadow, floodplain and barren are relatively low, while the ALT of alpine desert is relatively high. There is an obvious correspondence between ALT and the measured amplitude of seasonal deformation by our method in Figure 17. The ALT of the alpine meadow area is thinner than that of the alpine desert area, but the seasonal amplitude, which is related to soil moisture, is relatively high. The relationship between ALT and seasonal deformation is not obvious due to river erosion in the floodplain area, and the water content in the surrounding area is relatively high. 

## 6. Conclusions

In this paper, the NSBAS method with the seasonal deformation model provided new insights into complex permafrost investigation and deformation monitoring. This deformation model based on the accumulated degree days of freeze and thaw appears capable of accurately approximating and modeling the ground surface deformation of the frozen soil.

Our results show that the amplitude of seasonal deformation is between −62.15 mm and 11.5 mm, and the deformation rate ranged from −24.50 mm/yr to 5.00 mm/yr; the seasonal deformation and linear deformation rate are negatively correlated with elevation from Wudaoliang to Tuotuohe region. Additionally, the deformation results of six typical ground targets of the Beiluhe basin were analyzed in detail, and the seasonal deformation and linear deformation rates of the alpine meadow and floodplain were higher than those of alpine desert and barren. The QTR and QTH regions had a small subsidence trend, with maximums of 15.57 mm and 18.23 mm, respectively. The deformation results are also closely related to the ALT. 

In addition, the complete freeze–thaw cycle of the Beiluhe basin was analyzed by combining the daily air temperature data and the deformation map. The thawing process began from mid-April 2019 and ended in mid-September 2018 or 2019, and from the end of September 2018 or 2019, different typical ground targets began to freeze. Based on the TerraSAR-X and Sentinel-1 data, the time lags of the alpine desert are the longest, likely because the activity layer of the alpine desert is thicker than that of other regions. Based on the GPR data, there is a close relationship between seasonal deformation and the ALT. The seasonal deformation of the alpine meadow and the floodplain areas is relatively higher than the alpine desert area, with thinner ALT.

## Figures and Tables

**Table 1:** Detailed Information for SAR Data

Sensor	Temporal Coverage	Image Number	Orbit	Incidence Angle	Polarization	Range Pixel Spacing(m)	Azimuth Pixel Spacing(m)
Sentinel-1	08/07/2018–10/25/2019	35	Descending	34.46	VV	2.33	13.98
TerraSAR-X	12/15/2018–10/08/2019	9	Ascending	25.46	HH	0.454	0.167

**Table 2:** Amplitude of the seasonal deformation and linear deformation rate along the P1-P2 and Q1-Q2 profiles

Dataset	Typical Ground Targets	P1-P2 Profiles		Typical Ground Targets	Q1-Q2 Profiles	
		Amplitude of Seasonal Deformation (mm)	Linear Deformation Rate (mm/yr)		Amplitude of Seasonal Deformation (mm)	Linear Deformation Rate (mm/yr)
Sentinel-1	Alpine meadow	−48.53~−7.37	−23.73~1.45	alpine desert	−23.80~0.79	−13.70~0
	Alpine desert	−15.56~3.83	−14.93~0.56	barren	−20.33~−0.81	−12.13~0.46
	barren	−25.93~−2.41	−22.14~−7.69	floodplain	−38.17~−4.02	−19.12~−4.57
TerraSAR-X	Alpine meadow	−53.49~−5.54	−21.29~2.38	alpine desert	−20.87~−6.12	−15.76~−0.11
	Alpine desert	−19.63~−0.75	−18.65~−0.43	barren	−24.79~−0.80	−14.80~1.29
	barren	−45.84~−1.87	−21.43~−4.10	floodplain	−39.98~−6.05	−19.70~−1.04

**Table 3:** Time lags between the InSAR-observed displacement and the daily air temperature data

Dataset	Typical Ground Points	Date of the Maximum Uplift (Freezing Period)	Date of the Maximum Subsidence (Thawing Period)	Time Lags between the Maximum Uplift and the Minimum Air Temperature (01/20/2019)	Time Lags between the Maximum Subsidence and the Maximum Air Temperature (07/28/2019)
Sentinel-1	Point 1	04/04/2019	08/26/2019	74 days	53 days
	Point 2	04/16/2019	09/19/2019	86 days	65 days
	Point 3	03/23/2019	08/26/2019	74 days	29 days
	Point 4	03/11/2019	08/26/2019	62 days	29 days
TerraSAR-X	Point 1	04/04/2019	09/27/2019	74 days	61 days
	Point 2	04/26/2019	09/27/2019	96 days	61 days
	Point 3	03/02/2019	09/27/2019	41 days	61 days
	Point 4	03/02/2019	09/27/2019	41 days	61 days

**Table 4:** Permafrost deformation studies on the QTP using different deformation models

Study Area	InSAR Method	SAR Dataset	Observation Period	Amplitude of the Seasonal Displacement (mm)	Authors
Southern QTP	SBAS	Envisat ASAR	2007–2011	0.5–28	Li et al (2015)
Beiluhe	PSInSAR	TerraSAR-X	2014–2015	0–90	Wang et al (2016)
Northwestern QTP	NSBAS	Envisat ASAR	2003–2011	2.5–12	Daout et al (2017)
Same as this study	SBAS	ALOS-1 PALSAR	2007–2009	0–20	Jia et al (2017)
Northern QTP	PSInSAR	ALOS-1 PALSAR	2006–2011	−60–60	Chen et al (2018)
Same as this study	MT-InSAR	Sentinel-1	2017.11–2018.12	0–30	Zhang et al (2019)
South of Qinghai province	NSBAS	Sentinel-1 and TerraSAR-X	2018.8–2019.10	−62.50–11.50	This study
